# Scrotal MSSA Cellulitis Leading to Bacteremia with Septic Emboli to Lung and Brain w/o Evidence of Vegetations: Atypical Presentation Case Report

**DOI:** 10.51894/001c.144621

**Published:** 2025-09-30

**Authors:** Roman Permyakov

**Affiliations:** 1 Department of Internal Medicine Henry Ford Genesys

## 110

### BACKGROUND

A Staphylococcus aureus methicillin-sensitive and methicillin-resistant soft tissue infections have been known to lead to abscesses, bacteremia, endocarditis, osteomyelitis, and meningitis. Additionally, Staphylococcus aureus has been associated with Lemierre syndrome and septic emboli. S. aureus has great thrombotic capacity and can lead to vascular thrombosis and a subsequent septic emboli or secondary sites of infection. This is a case of MSSA soft tissue infection that appears to spread hematogenously, leading to multiple sequalae that are typically seen in either thrombophlebitis or infectious endocarditis but rarely present simultaneously.

### CASE

A 58 year old male with history of a recent CVA presents with embolic strokes in the brain with multiple peripherally predominant nodular densities scattered throughout lungs with few nodules demonstrating central cavitation. Found to have no evidence of cardiac vegetations on transthoracic or transesophageal echocardiography, no evidence of thromboembolisms or thrombophlebitis. Patient was found to have a soft tissue infection of the scrotum with MSSA as well as MSSA bacteremia without abscess or necrotizing fasciitis. No other signs of aggregated infection identified. This is an uncommon presentation of Staphylococcus aureus methicillin-sensitive soft tissue infection leading to septic emboli and ischemic stroke without evidence of alternative source.

### CONCLUSION

This case report serves to demonstrate the importance of maintaining a broad differential when presented with embolic strokes. The case was complicated by new onset atrial fibrillation and a scrotal soft tissue infection. Atrial fibrillation, Loeffler’s endocarditis, Infective endocarditis, thrombophlebitis, pyomyositis, osteomyelitis, abscesses, and necrotizing fasciitis were among causes of embolic thrombi that were considered. Further investigations could have included mechanical thrombectomy, or coagulation disorders although resources and size of emboli limited these options. The main points of this case are, thorough workup, broad differentials, and close follow up in outpatient setting with primary care and specialists such as Cardiology and Hematology for further work up.

Limitations include, no genetic analysis of the MSSA, no toxin screening. In some cases, clots can be biopsied through mechanical thrombectomy in order to determine a clot as infectious vs noninfectious but no samples were obtained due to size of emboli.

